# In the national epidemiological bulletins – a selection from recent issues

**DOI:** 10.2807/1560-7917.ES.2019.24.39.1909261

**Published:** 2019-09-26

**Authors:** Eurosurveillance editorial team

**Affiliations:** 1 European Centre for Disease Prevention and Control (ECDC), Stockholm, Sweden

**Keywords:** infectious diseases, public health, Europe

**Healthcare-associated infections and antibiotic resistance**

 

Suspected and laboratory confirmed reported norovirus outbreaks in hospitals (England) and norovirus laboratory reports (England and Wales): weeks 31 to 35, 2019Health Protection Report; 13(32)

16 September 2019, United Kingdom

https://assets.publishing.service.gov.uk/government/uploads/system/uploads/attachment_data/file/831662/hpr3219_noro.pdf

 

Characteristics, occurrence and spread of vancomycin-resistant enterococci in Germany – Update 2017/2018

Epidemiologisches Bulletin 35/2019

29 August 2019, Germany

https://www.rki.de/DE/Content/Infekt/EpidBull/Archiv/2019/Ausgaben/35_19.pdf?__blob=publicationFile

 

**Food- and waterborne diseases**

 

Routine reports of gastrointestinal infections in humans, England and Wales: July and August 2019

Health Protection Report; 13(32)

19 September 2019, United Kingdom

https://assets.publishing.service.gov.uk/government/uploads/system/uploads/attachment_data/file/831639/hpr3219_GIs.pdf

 

**Hepatitides**

 

Epidemiology of hepatitis A in Ireland, 2018

Epi-Insight 2019;20(9)

September 2019, Ireland

http://ndsc.newsweaver.ie/epiinsight/5vwalp0t10e10gkzp9yxn5?a=2&p=55562857&t=17517804

 

Laboratory reports of hepatitis A infections in England and Wales, 2018

Health Protection Report; 13(28)

9 August 2019, United Kingdom

https://assets.publishing.service.gov.uk/government/uploads/system/uploads/attachment_data/file/824201/hpr2819_HAV18.pdf

 

Hepatitis C reporting according to the Infection Protection Act, 2016-2018: effects of changes to case definition and mandatory notification

Epidemiologisches Bulletin 30/2019

25 July 2019, Germany

https://www.rki.de/DE/Content/Infekt/EpidBull/Archiv/2019/Ausgaben/30_19.pdf?__blob=publicationFile

 

Viral hepatitis B and D in 2018

Epidemiologisches Bulletin 29/2019

18 July 2019, Germany

https://www.rki.de/DE/Content/Infekt/EpidBull/Archiv/2019/Ausgaben/29_19.pdf?__blob=publicationFile

 

**Vaccine-preventable diseases**

 

Tuberculosis in 2018

EPI-NEWS 37, 2019

20 September 2019, Denmark

https://en.ssi.dk/news/epi-news/2019/no-37---2019

 

Evolution of human papillomavirus vaccination coverage in France – 2008-2018

Bulletin épidémiologique hebdomadaire, 22-23/2019

17 September2019, France

http://beh.santepubliquefrance.fr/beh/2019/22-23/2019_22-23_3.html

 

High incidence of whooping cough

EPI-NEWS 28/33, 2019

21 August 2019, Denmark

https://en.ssi.dk/news/epi-news/2019/no-28-33---2019

 

Mumps outbreak in Ireland 2019

Epi-Insight 2019;20(8)

August 2019, Ireland

http://ndsc.newsweaver.ie/epiinsight/l44oejj60op10gkzp9yxn5?a=1&p=55435965&t=17517774

 

Invasive pneumococcal disease and the impact of vaccines in Ireland, 2008-2018

Epi-Insight 2019;20(8)

August 2019, Ireland

http://ndsc.newsweaver.ie/epiinsight/1fu7nip7a4o10gkzp9yxn5?a=1&p=55435965&t=17517774

 

HPV vaccination for boys

EPI-NEWS 24/25, 2019

3 July 2019, Denmark

https://en.ssi.dk/news/epi-news/2019/no-24-25---2019

 

Laboratory confirmed cases of invasive meningococcal infection (England): January to March 2019

Health Protection Report; 13(22)

1 July 2019, United Kingdom

https://assets.publishing.service.gov.uk/government/uploads/system/uploads/attachment_data/file/813291/hpr2219_IMD.pdf

 

Laboratory confirmed cases of pertussis (England): January to March 2019

Health Protection Report; 13(22)

1 July 2019, United Kingdom

https://assets.publishing.service.gov.uk/government/uploads/system/uploads/attachment_data/file/813292/hpr2219_prtsss.pdf

 

Introduction of MenACWY vaccine for students starting secondary school in 2019

Epi-Insight 2019;20(7)

July 2019, Ireland

http://ndsc.newsweaver.ie/epiinsight/12xqmkrpcvp10gkzp9yxn5?a=1&p=55256146&t=17517774

 

Vaccine-preventable diseases

Vlaams Infectieziektebulletin

2/2019, Belgium

https://www.zorg-en-gezondheid.be/sites/default/files/atoms/files/VIB%202019-2_3%20Infectieziekten%20te%20voorkomen%20door%20vaccinatie.pdf

 

**Respiratory diseases**

 

Improved uptake of seasonal influenza vaccine by healthcare workers

Epi-Insight 2019;20(9)

September 2019, Ireland

http://ndsc.newsweaver.ie/epiinsight/2ibb6fawkni10gkzp9yxn5?a=2&p=55562857&t=17517804

 

Integrated influenza surveillance in Italy: results of the 2018-19 season

Not Ist Super Sanità 2019;32(7-8)

July-August 2019, Italy

http://old.iss.it/binary/publ/cont/BEN_LUGLIO_AGOSTO.pdf

 

Outbreak of legionellosis in outpatients in Bremen 2015 and 2016 – experiences, results, decisions

Epidemiologisches Bulletin 28/2019

11 July 2019, Germany

https://www.rki.de/DE/Content/Infekt/EpidBull/Archiv/2019/Ausgaben/28_19.pdf?__blob=publicationFile

 

**Sexually transmitted diseases**

 

Gonorrhoea diagnosed in 2018

EPI-NEWS 35/36, 2019

16 September 2019, Denmark

https://en.ssi.dk/news/epi-news/2019/no-35-36---2019

 

Syphilis 2018

EPI-NEWS 34, 2019

6 September 2019, Denmark

https://en.ssi.dk/news/epi-news/2019/no-34---2019

 

Gonorrhoea with a highly azithromycin-resistant pathogen in Germany

Epidemiologisches Bulletin 32-33/2019

8 August 2019, Germany

https://www.rki.de/DE/Content/Infekt/EpidBull/Archiv/2019/Ausgaben/32-33_19.pdf?__blob=publicationFile

 

Sexually transmitted diseases

Vlaams Infectieziektebulletin

2/2019, Belgium

https://www.zorg-en-gezondheid.be/sites/default/files/atoms/files/VIB%202019-2_2%20Seksueel%20overdraagbare%20aandoeningen.pdf

 

**Zoonoses and vector-borne diseases**

 

Common animal-associated infections quarterly report (England and Wales): second quarter 2019

Health Protection Report; 13(28)

9 August 2019, United Kingdom

https://assets.publishing.service.gov.uk/government/uploads/system/uploads/attachment_data/file/824211/hpr2819_zoos.pdf

 

Special issue: Arboviruses: Surveillance data to anticipate their control

Bulletin épidémiologique hebdomadaire, 19-20/2019

9 July 2019, France

http://beh.santepubliquefrance.fr/beh/2019/19-20/index.html

 

**Other**

 

Recommendations of the Vaccine Standards Commission at the Robert Koch Institute – 2019/2020

Epidemiologisches Bulletin 34/2019

22 August 2019, Germany

https://www.rki.de/DE/Content/Infekt/EpidBull/Archiv/2019/Ausgaben/34_19.pdf?__blob=publicationFile

 

Creutzfeldt-Jakob disease (CJD) surveillance: biannual updates

Health Protection Report; 13(28)

9 August 2019, United Kingdom

https://assets.publishing.service.gov.uk/government/uploads/system/uploads/attachment_data/file/824206/hpr2819_CJD.pdf

 

Laboratory surveillance of candidaemia in England, Wales and Northern Ireland: 2018

Health Protection Report; 13(25)

19 July 2019, United Kingdom

https://assets.publishing.service.gov.uk/government/uploads/system/uploads/attachment_data/file/818902/hpr2519_cnddm18.pdf

